# Evaluation of ciliary cleft changes after phacoemulsification using ultrasound biomicroscopy in dogs with cataracts

**DOI:** 10.3389/fvets.2023.1247127

**Published:** 2023-11-15

**Authors:** Donghee Kim, Yeong-Seok Goh, Hyemin Kim, Sang-Eun Park, Jiyi Hwang, Nanyoung Kang, Ji Seung Jung, Kyung-Mee Park

**Affiliations:** Laboratory of Veterinary Surgery and Ophthalmology, College of Veterinary Medicine, Chungbuk National University, Cheongju, Republic of Korea

**Keywords:** ciliary cleft, iridocorneal angle, cataract, phacoemulsification, glaucoma, ultrasound biomicroscopy, aqueous humor, trabecular meshwork

## Abstract

**Introduction:**

Glaucoma is one of the most serious complications that causes irreversible blindness after phacoemulsification in dogs; however, a clear mechanism has not been elucidated. This study aimed to analyse the possible anatomical factors associated with glaucoma after phacoemulsification using parameters that reflect the anatomical characteristics of dogs.

**Materials and methods:**

A total of 69 eyes of 48 dogs were included in this study. The patients were divided into three groups: normal eye (*n* = 18), cataract (*n* = 39), and post-phacoemulsification for at least 2 months after surgery (post-phaco, *n* = 12). For further analysis, the dogs were subdivided into two groups according to cataract stage: phacoemulsification non-candidate and candidate groups. Non-cataracts and incipient cataracts were categorized into the non-candidate group, whereas immature and mature cataracts were categorized into the candidate group. Measurements of the ciliary cleft parameters, including the area of the ciliary cleft (CCA), length of the ciliary cleft (CCL), width of the ciliary cleft (CCW), iridocorneal angle, and angle opening distance, were obtained using ultrasound biomicroscopy.

**Results:**

CCA, CCL, and CCW were significantly higher in the candidate group than in the non-candidate group. CCA, CCL, and CCW were significantly reduced in the post-phaco group compared to those in the cataract group. Based on these results, we found that the ciliary cleft expanded in cataract-affected eyes and narrowed after phacoemulsification. This may indicate that the space between the trabecular meshworks became narrower, potentially leading to an increase in the resistance of the aqueous humor.

**Conclusion:**

A narrowed ciliary cleft after phacoemulsification may be an anatomical factor associated with glaucoma.

## Graphical Abstract

Illustration of ciliary cleft changes after phacoemulsification in cataract dogs. **(A)** Normal eye group, **(B)** cataract eye; the ciliary cleft expanded in the cataract group compared to the normal eye group due to the tension applied to the zonule by the cataract-affected lens. **(C)** Post-phaco eye; In contrast, in the post-phaco group, phacoemulsification removed the lens material and reduced the tension of the zonule, causing the ciliary cleft to subsequently narrow. 
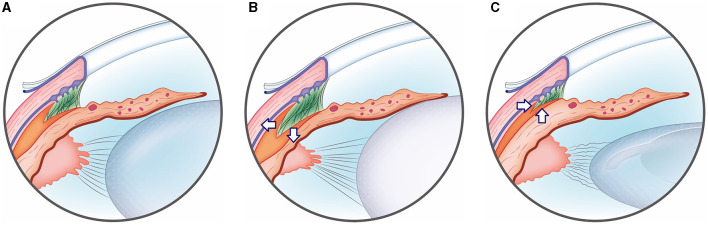

## 1 Introduction

Glaucoma is a potentially serious complication of phacoemulsification that can lead to irreversible vision loss and severe pain in dogs (1, 2). In a previous study involving 179 eyes in 103 dogs, the incidence of glaucoma as a complication of phacoemulsification was 6.7% (12/179 eyes) during an average follow-up period of 302 days. At the final examination, 75.0% (9/12 eyes) of the glaucoma cases were blind, 8.3% (1/12 eyes) had reduced vision, and 16.7% (2/12 eyes) had preserved visual function (1). Although risk factors such as breed, age, cataract stage, uveitis, and iridocorneal angle (ICA) have been demonstrated in post-operative hypertension (POH) or glaucoma after phacoemulsification, the fundamental anatomical mechanism for inducing glaucoma has not been demonstrated in dogs (2–4).

The ciliary cleft (CC) is located in the peripheral circumferential space anterior to the iris-ciliary body complex, which is part of the aqueous humor (AH) pathway. The CC is a triangular space in cross-section composed of the trabecular meshwork (TM). The space between the TM is called Fontana's space. In this space, the sclera is external, the anterior pars plicata of the ciliary body and iris root are internal, the ciliary muscle and matrix are posterior, and the pectinate ligaments are anterior (5, 6). Therefore, relaxation or contraction of the ciliary muscle can influence the anatomical morphology of the CC and, consequently, the resistance to AH outflow (5).

Ultrasound biomicroscopy (UBM) is a useful tool for visualizing structures in the anterior chamber with a high tissue-penetration ability (7). Compared to regular ultrasound devices, such as A-scan or B-scan, which operate at frequencies of 10 MHz, UBM uses a much higher frequency transducer, ranging from 50 to 100 MHz. UBM provides cross-sectional images and quantitative assessment of the anterior ocular segment, such as the ICA and anterior chamber depth (8). Moreover, it can visualize the posterior pigmented layers of the iris, such as the ciliary body and muscles (9).

UBM has been used in humans to evaluate the trabecular iris angle (TIA), which is equivalent to the ICA in veterinary medicine, and the angle-opening distance (AOD) before and after phacoemulsification. In this study, both the TIA and AOD increased postoperatively (10). Similarly, the AOD and ICA before and after surgery were evaluated as risk factors for POH after phacoemulsification in dogs (11). However, unlike humans, no significant changes in these factors after surgery were observed in dogs. Therefore, anatomical CC changes that cause glaucoma after phacoemulsification have not yet been elucidated (11).

Furthermore, due to anatomical differences in the position of the trabecular meshwork, between humans and dogs, it is not appropriate to utilize ICA or anterior chamber angle opening distance (AOD), commonly used in human studies, to investigate the potential causes of glaucoma following cataract surgery in dogs (12, 13).

The aim of this study was to describe the anatomical characteristics of the CC and ICA in dogs who have undergone phacoemulsification and compare them with dogs who have not undergone the procedure. By examining these parameters, our objective was to investigate the differences in the AH pathway in patients with cataracts or those who have undergone phacoemulsification. We aimed to provide valuable insights into the anatomical features of the CC and ICA in dogs after phacoemulsification, which can serve as a foundation for future research in this field. To the best of our knowledge, this is the first study to utilize UBM to investigate the anatomical characteristics of the aqueous humor pathway in dogs undergoing cataract surgery.

## 2 Materials and methods

### 2.1 Collection of clinical information

This was a prospective study conducted on 69 eyes (48 dogs). The clinical information of the dogs was collected from the Veterinary Teaching Hospital of Chungbuk National University in Korea between 29th August 2018 and 20th September 2022. This study was approved by the Institutional Animal Care and Use Committee (CBNUA-1700-22-02). All dogs enrolled in this study underwent a comprehensive ophthalmological examination performed by a veterinary faculty (KM Park) or by veterinarians specializing in ophthalmology (other authors). The examination included slit-lamp biomicroscopy (MW50D, SHIGIYA, Hiroshima, Japan), Schirmer tear test (Schirmer Tear Flow Strips, GuldenOphthalmics, PA), menace response, pupillary light reflex, dazzle reflex, rebound tonometry (TonoVet plus^®^, icare, Vantaa, Finland), gonioscopy (Ocular Koeppe Diagnostic Lenses, Ocular Instruments.inc, Bellevue, WA), indirect ophthalmoscopy (Pan Retinal^®^ 2.2, VOLK, OH), and UBM (VuPAD^®^, Sonomed Escalon, Lake Success, NY).

Cataract stages in this study are classified as incipient, immature, and mature. Incipient cataracts present with early focal opacity with minimal vision impact and no detectable lens-induced uveitis (LIU). Immature cataracts exhibit extended opacity affecting most of the lens, compromising vision but still permitting some visual capability. Finally, mature cataracts lead to total lens opacity, causing functional blindness and often associated with LIU. Recognizing these stages is crucial for timely and appropriate interventions (14).

#### 2.1.1 Patient groups

The dogs were divided into three groups: (a) normal eye, (b) cataract, and (c) post-phacoemulsification (post-phaco). For further analysis, the normal eye and cataract groups were subdivided again into two groups: (a) phacoemulsification candidate and (b) phacoemulsification non-candidate groups (15–20). The phacoemulsification candidate group consisted of immature and mature cataracts, whereas the non-candidate group included normal eyes and incipient cataracts.

#### 2.1.2 Inclusion criteria

Patients with hereditary or senile cataracts were eligible for inclusion. Only patients who were followed up for at least 2 months (mean: 662 ± 465 days; range: 61–1,365 days) after phacoemulsification were included in the post-phaco analysis.

#### 2.1.3 Exclusion criteria

In this study, patients with a low IOP were excluded to eliminate the possibility of unseen low-grade inflammation affecting the results. Patients with hypermature cataract were excluded from the study population due to potential distortions in the CC caused by factors such as lens-induced uveitis. Patients presenting with conditions such as uveitis, glaucoma, or hemorrhage, other than cataracts, were also excluded. Additionally, patients with secondary cataracts resulting from diabetes or trauma were not included. Eyes exhibiting a closed Iridocorneal Angle (ICA) during gonioscopy were excluded from the study. Furthermore, patients who had undergone implantation of a Capsular Tension Ring (CTR) (an-CTR-12, An-vision, Hennigsdorf, Germany) were not included in the analysis.

### 2.2 UBM examination

UBM was performed at the time of ophthalmic examination in normal and cataractous eyes. For post-phaco eyes, UBM was performed at least 2 months after cataract surgery. With the dogs in a sitting position, all participating patients underwent pupil dilation through the topical application of 0.5% tropicamide, administered as one drop every 10 min for a duration ranging from 15 to 30 min. This ensured that pupils were adequately dilated at the time of the UBM examination. Additionally, one drop of topical anesthesia, 0.5% proparacaine hydrochloride (Alcaine^®^ Alcon), was administered. The palpebral fissure was manually opened, and the eye was examined using a transducer placed perpendicular to the corneoscleral limbus in the dorsal quadrant.

**Figure 1:**
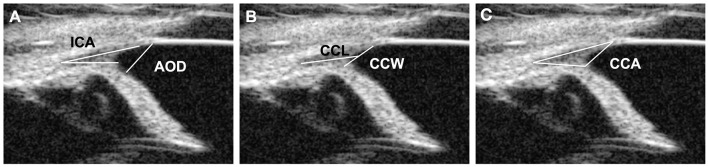
UBM measurement method. **(A)** Measurement of the angle-opening distance (AOD) and the iridocorneal angle (ICA). The ICA represents the peripheral circumference of the anterior chamber, where the sclera, cornea, and base of the iris converge. The AOD was measured from the end of Descemet's membrane, which was used as an anatomical landmark to the anterior iris surface. **(B)** Measurements of the ciliary cleft width (CCW) and the ciliary cleft length (CCL). The CCW was measured by determining the distance between the root of the iris and the corneosclera. The CCL was measured by determining the distance between the angle recess and the middle point of the CCW. **(C)** Measurements of the ciliary cleft area (CCA). The CCA was measured as the area surrounded by the ciliary cleft.

Five CC parameters were evaluated for each eye according to the previous research: (a) geometric iridocorneal angle (ICA) (i.e., angle of the plane of the iris root and posterior corneoscleral limbus), (b) angle-opening distance (AOD), (c) width of the entrance of the CC (CCW), (d) length of the CC (CCL), and (e) manually measured area of the CC (CCA) (Figure 1) (5, 6, 11, 21, 22). At least three different images were used for the measurements, and the average values were obtained. The measurements of these parameters were performed using the built-in software of the UBM.

To avoid bias, the UBM examinations were conducted by one set of individuals, while the measurements using the UBM's built-in software were conducted by a different individual, DH Kim, who was masked to the patient groups. DH Kim was unaware of which patient's images were being analyzed to ensure objectivity in the measurements.

### 2.3 Cataract surgery: pre and post-surgical management

Pre-operatively, topical ophthalmic antibiotics (moxifloxacin, Vigamox^®^, NOVARTIS, East Hanover, NJ or ofloxacin, OcuFlox^®^, Samil, Seoul, Korea), non-steroidal anti-inflammatory drugs (flurbiprofen, flurbiprofen^®^, Bausch & Lomb, Bridgewater, NJ, or bromfenac, Bronuck^®^, TAEJOON PHARM, Seoul, Korea), and steroids (neomycin-polymyxin B sulfate-dexamethasone Maxitrol^®^, NOVARTIS, East Hanover, NJ or prednisolone acetate 1%, Pred-Forte^®^, Allergan, North Chicago, IL) were administered q8h, q12-24h, and q8h, respectively, for 5 days. Amoxicillin-clavulanate (AMOCLA^®^, KUHNIL PHARM, Cheonan, Korea), famotidine (Famotidine, Nelson, Eumsung, Korea), and carprofen (ASHICARP^®^, ALS, Mumbai, India) were administered for 5 days preoperatively. Three to four hours before surgery, the following ophthalmic solution was administered to each eye in three to four cycles, with 5–10 min intervals between cycles: moxifloxacin or ofloxacin, flurbiprofen or bromfenac, neomycin-polymyxin B sulfate-dexamethasone or prednisolone, tropicamide, and atropine (Atropine^®^, Alcon).

One veterinary faculty (KM Park) and two veterinarians specializing in ophthalmology (JH Ahn and JW Park) performed the phacoemulsification. Three phacoemulsification machines were used (Stellaris PC, Bausch & Lomb Inc.; Pulsar Minimal Stress, OPTIKON, Roma, Italy; or Sovereign Compact Phaco Machine, AMO, Andrew Place Santa Ana, CA). Foldable acrylic IOLs (Loki^®^, CRISTALENS, Lannion, France) with a power of +41.0 diopters and diameter of 12.00–14.00 mm were implanted through a 3.0 mm stepped clear corneal incision. The irrigation fluid was a balanced salt solution (BSS Plus; Alcon or Opticosol solution; Reyon Pharm, Seoul, Korea) with epinephrine (1 mg/mL; DAIHAN, Seoul, Korea) and heparin (5,000 units/mL; JW, Seoul, Korea) additives. During surgery, ophthalmic viscoelastic material (Kukje Hyaluronate-I Inj; KUKJE, Geonggi, Korea) was used and removed via irrigation/aspiration (I/A) at the end of surgery. After determining that all viscoelastic substances were removed, I/A was performed for 30 s for complete washing.

The corneal incision was completely closed with 8-0 polyglactin (Vicryl, Ethicon, Somerville, NJ) or 8-0 nylon (Usiol, Lexington, KY) sutures using a spatula needle in a simple interrupted pattern. Subconjunctival dexamethasone (2 mg/eye; JEIL, Seoul, Korea) and cefazolin (50 mg/eye; Chongkundang) were administered at the end of the surgery. Intraocular pressure (IOP) was measured immediately after surgery and every 4 for 48 h postoperatively.

Topical steroids used included prednisolone acetate 1% (q6h for 2 weeks, then tapered for 2–4 weeks) or neomycin-polymyxin B sulfate-dexamethasone (q6h for 2 weeks, then tapered for 4 weeks). Moxifoxacin or ofloxacin was used as a topical antibiotic (q6h for 2 weeks and q8-12h for 2–3 weeks). Systemic antibiotics (amoxicillin-clavulanate PO q12h) were administered postoperatively for 2–3 weeks. Systemic steroids (prednisolone, 0.5 mg/kg PO q12h for 1–2 week, then tapered for 1–4 weeks) were administered postoperatively.

### 2.4 Statistical analysis

Statistical analyses were conducted using SPSS software (version 17.0; SPSS Inc., Chicago, IL, USA). The normality of the data was assessed using Shapiro-Wilk analysis. ANOVA was employed to compare the normal eye, cataract, and post-operative groups, followed by *post-hoc* analyses using Fisher's exact test. A *t*-test was utilized to compare the non-candidate and candidate groups. Statistical significance was defined as ^*^P < 0.05, ^**^P < 0.01, ^***^P < 0.001, and ^****^P < 0.0001. Asterisks were used in the figures for convenience, while *p*-values were reported in the text.

## 3 Results

### 3.1 Canine characteristics

#### 3.1.1 Normal eye group

Among the 18 normal eyes of the nine dogs, there were eight purebred dogs and one mixed-breed dog. The most common breeds were the Poodle, Shih Tzu, Maltese, and Chihuahua. There were five male and four female dogs. The mean age of these dogs was 9.53 years (range, 1.5–15 years), and their mean weight was 5.17 ± 2.36 kg (range, 2.85–9.80 kg).

#### 3.1.2 Cataract group

Among the 39 cataractous eyes of 24 dogs, 17 were incipient, 16 were immature, and six were mature. This group included 24 purebred dogs, the most common of which were Poodle, Bichon Frise, and Shih-tzu. This study included 10 males and 14 females. The mean age of these dogs was 8.45 ± 2.89 years (range, 2.2–13.0 years), and their mean weight was 4.94 ± 2.45 kg (range, 1.80–9.80 kg).

#### 3.1.3 Post-phaco group

The post-phaco group included 12 eyes of 10 dogs. Patients with glaucomatous eyes were excluded. Before surgery, the cataract stages were defined as five immature and seven mature eyes. There were 10 purebred dogs. The most common breeds were Maltese, Bichon Frise, and Poodle. There were three males and seven females. The mean age of these dogs was 9.58 ± 2.71 years (range, 5.28–12.4 years), and their mean weight was 4.50 ± 1.94 kg (range, 2.4–8.1 kg). There were no significant differences in age, weight, or breeding between the groups.

### 3.2 CC parameter changes according to cataract maturation

**Figure 2:**
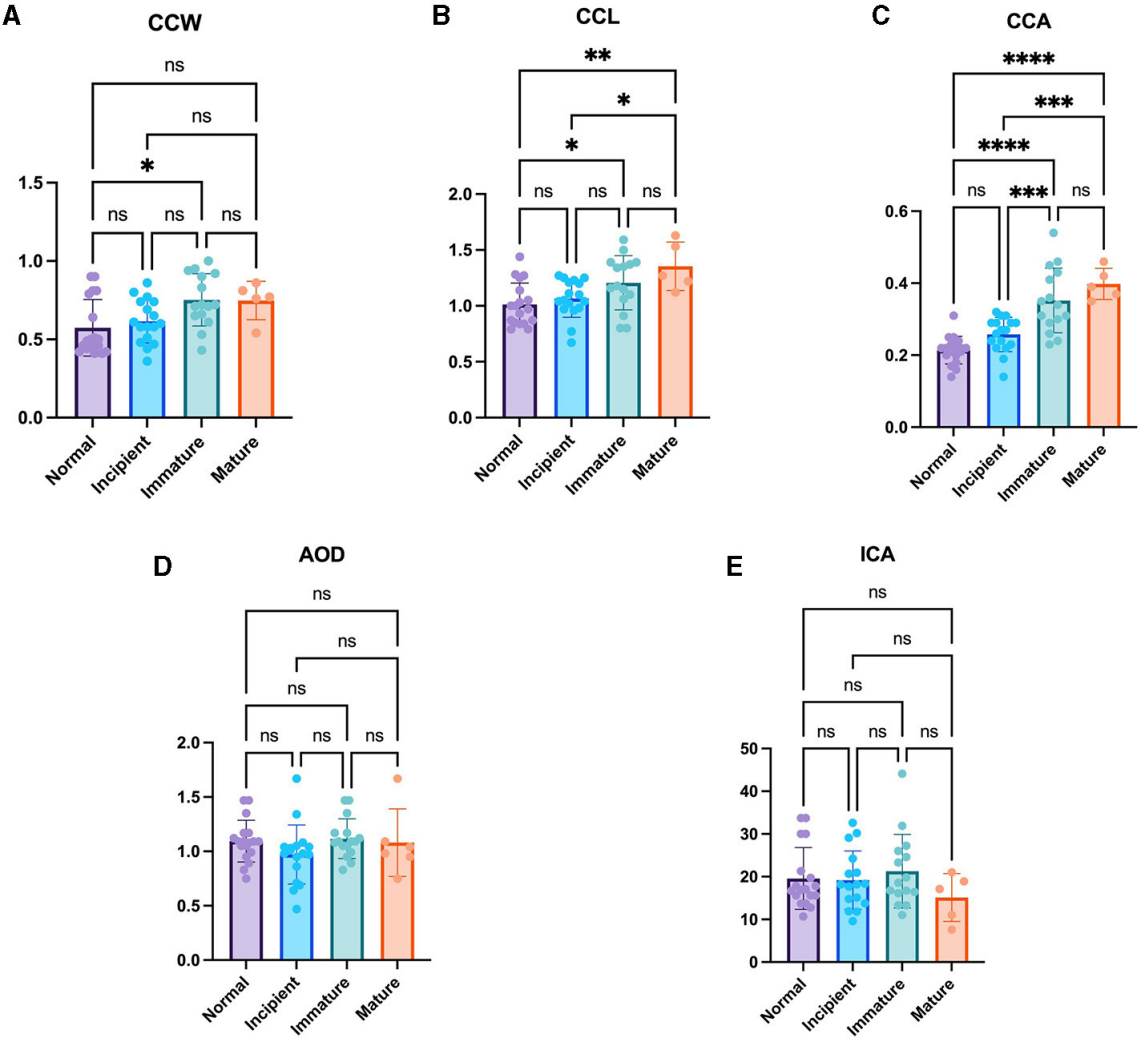
Comparison of the normal eye, incipient, immature and mature cataract. **(A)** CCW, **(B)** CCL, **(C)** CCA, **(D)** AOD, and **(E)** ICA in the normal eye, incipient, immature and mature cataract. There were significant differences in CCW between normal and immature eyes. There were significant differences in CCL between normal and immature eyes, normal and mature eyes, as well as incipient and mature eyes. There were significant differences in CCA between normal and immature eyes, normal and mature eyes, incipient and immature eyes as well as incipient and immature eyes. However, the AOD and ICA were not significantly different between the groups. *Indicates *P* < 0.05, **represents *P* < 0.01, ***denotes *P* < 0.001, and ****signifies *P* < 0.0001.

As the cataract stage progressed, the values of CC parameters, including CCW, CCL, and CCA, tended to increase. Specifically, CCW exhibited a significantly larger value in the immature cataract (0.75 ± 0.17 mm) compared to the normal eye (0.57 ± 0.18 mm) (*P* < 0.05). Furthermore, CCL demonstrated significantly higher values in both the immature cataract (1.21 ± 0.24 mm) and mature cataract (1.32 ± 0.22 mm) compared to the normal eye (1.01 ± 0.19 mm) (*P* < 0.05, *P* < 0.01, respectively), and significantly higher values in the mature cataract (1.32 ± 0.22 mm) compared to the incipient cataract (1.07 ± 0.17 mm) (*P* < 0.05). In the case of CCA, both the immature cataract (0.35 ± 0.09 mm^2^) and mature cataract (0.40 ± 0.04 mm^2^) showed significantly higher values than the normal eye (0.21 ± 0.04 mm^2^) (*P* < 0.0001, *P* < 0.0001, respectively), and both the immature cataract (0.35 ± 0.09 mm^2^) and mature cataract (0.40 ± 0.04 mm^2^) showed significantly higher values than the incipient cataract (0.26 ± 0.05 mm^2^) (*P* < 0.001, *P* < 0.001, respectively). However, statistically significant differences could not be observed in the comparison of ICA and AOD across all cataract stages (Figure 2).

### 3.3 CC changes in phacoemulsification candidate and non-candidate groups

**Figure 3:**
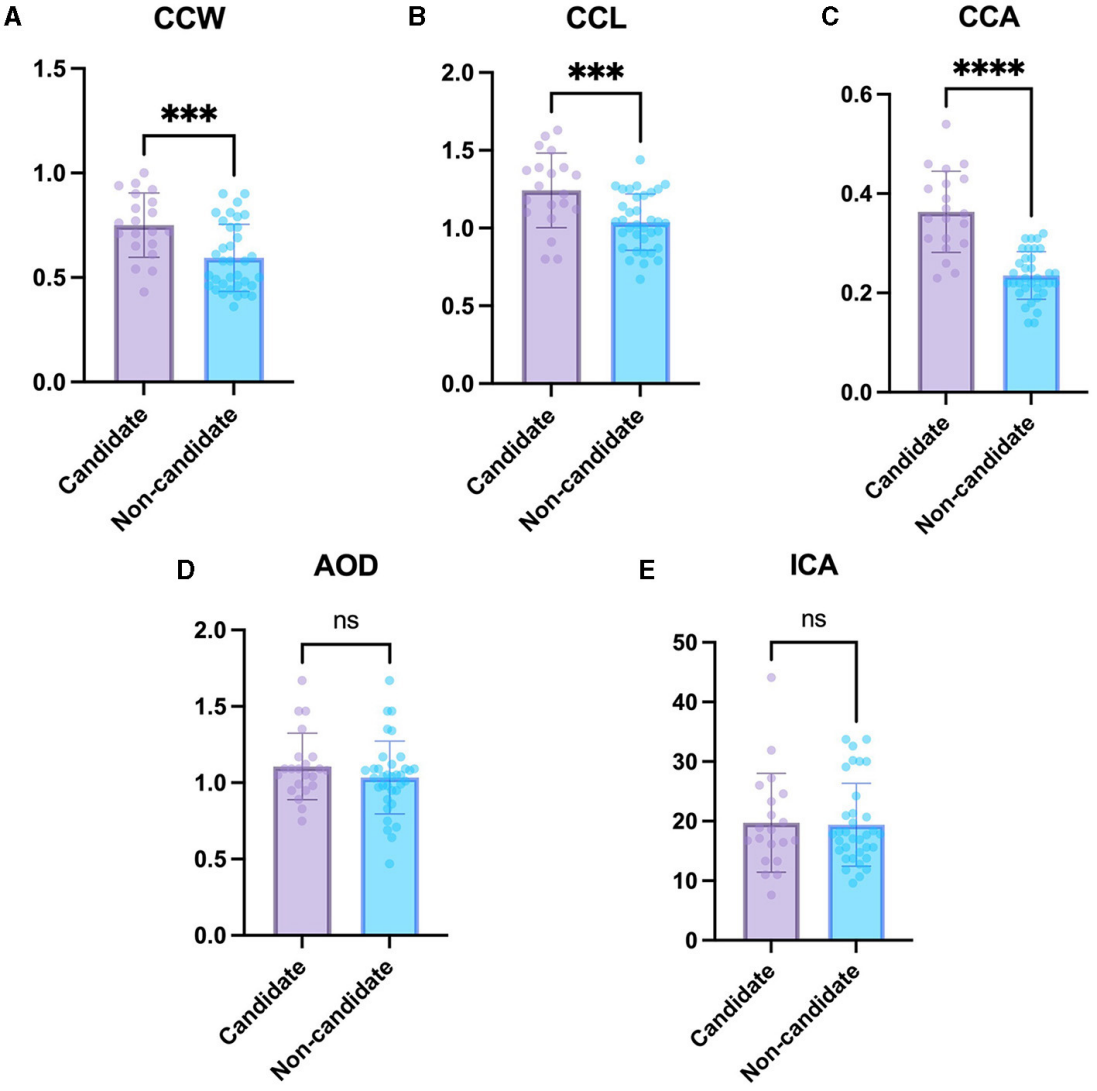
Comparison of the non-candidate and candidate groups. **(A)** CCW, **(B)** CCL, **(C)** CCA, **(D)** AOD, and **(E)** ICA in the candidate group compared with non-candidate group. The CCW, CCL, and CCA were significantly larger in the candidate group than in the non-candidate group. However, the AOD and ICA were not significantly different between the groups. *Indicates *P* < 0.05, **represents *P* < 0.01, ***denotes *P* < 0.001, and ****signifies *P* < 0.0001.

The CCW, CCL, and CCA were significantly larger in the phacoemulsification candidate group including immature and mature cataracts (0.75 ± 0.15 mm, 1.24 ± 0.24 mm, and 0.36 ± 0.08 mm^2^, respectively) than in the non-candidate group including normal eyes and incipient cataracts (0.59 ± 0.16 mm, 1.04 ± 0.18 mm, and 0.24 ± 0.05 mm^2^, respectively) (*P* < 0.01, *P* < 0.001, *P* < 0.0001, respectively) (Figures 3A–C). This suggests that the CC is larger in the candidate group compared to that in the non-candidate group.

The difference in AOD between the non-candidate group (1.04 ± 0.24 mm) and candidate group (1.11 ± 0.22 mm) was not statistically significant (*P* = 0.26). In addition, the difference in ICA between the non-candidate group (19.40 ± 6.95°) and candidate group (19.74 ± 8.30°) was not statistically significant (*P* = 0.87) (Figures 3D, E).

### 3.4 CC changes in normal eye, cataract, and post- phaco group

**Figure 4:**
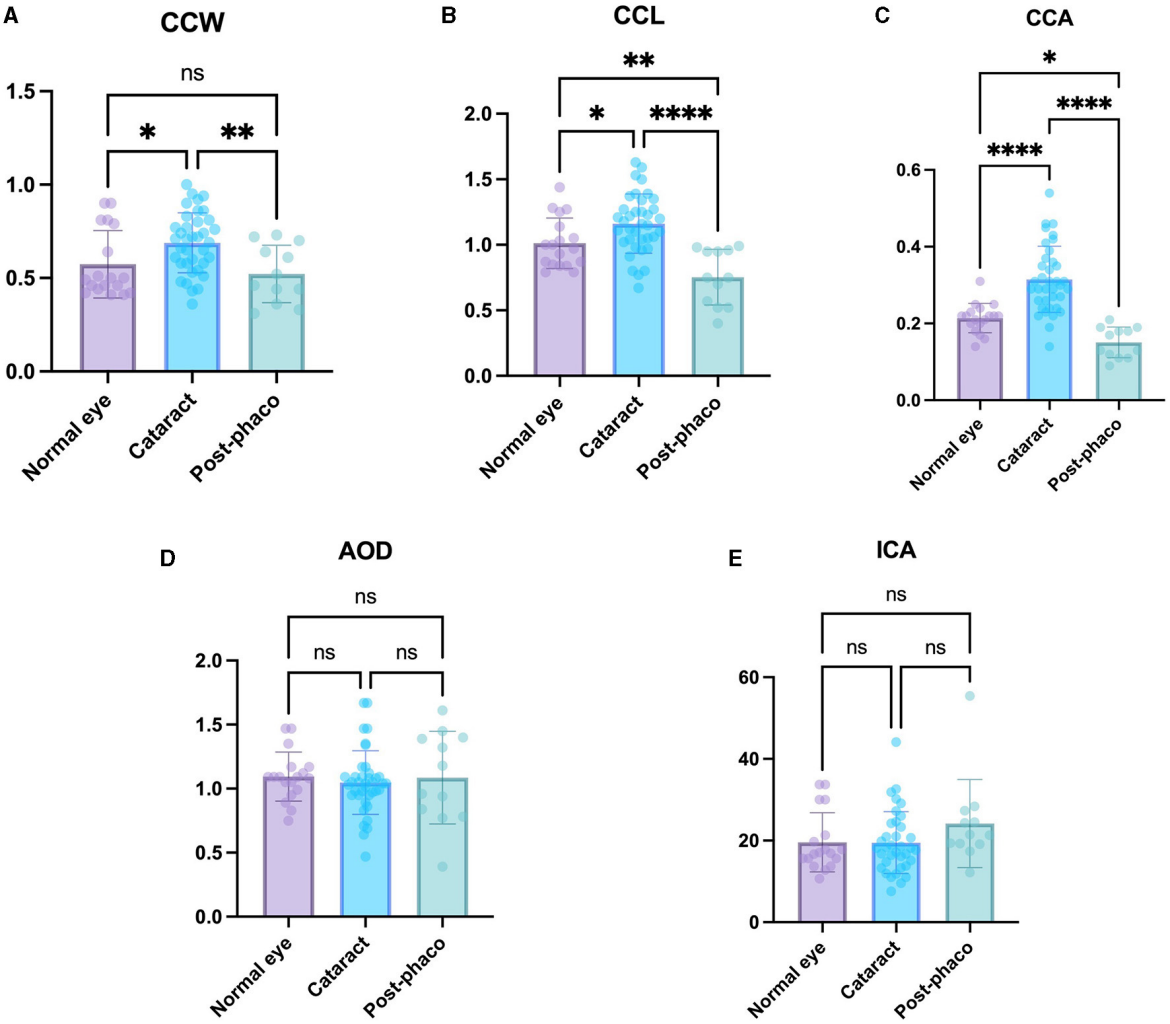
Comparison of the normal, cataract, and post-phaco groups. Comparison of **(A)** CCW, **(B)** CCL, **(C)** CCA, **(D)** AOD, and **(E)** ICA in the normal eye, cataract, and post-phaco groups. The CCW, CCL, and CCA are larger with cataract formation. The CCW, CCL, and CCA are smaller after phacoemulsification. *Indicates *P* < 0.05, **represents *P* < 0.01, ***denotes *P* < 0.001, and ****signifies *P* < 0.0001.

The cataract group exhibited a significant larger CCW, CCL, and CCA (0.69 ± 0.16 mm, 1.16 ± 0.23 mm, and 0.31 ± 0.09 mm^2^, respectively) compared to the normal eye group (0.57 ± 0.18 mm, 1.01 ± 0.19 mm, and 0.21 ± 0.04 mm^2^, respectively) (*P* < 0.05, *P* < 0.05, *P* < 0.0001, respectively) (Figures 4A–C). These findings suggest that the CC parameter became larger as the cataract progressed.

The post-phaco group demonstrated a significant smaller CCW, CCL, and CCA (0.52 ± 0.15 mm, 0.75 ± 0.21 mm, and 0.15 ± 0.04 mm^2^) compared to the cataract group (0.69 ± 0.16 mm, 1.16 ± 0.23 mm, and 0.31 ± 0.09 mm^2^, respectively) (*P* < 0.01, *P* < 0.0001, *P* < 0.0001, respectively) (Figures 4A–C). These results suggest that the CC parameter showed a relatively small value in the post-phaco group.

The CCL and CCA were significantly larger in the normal eye group (1.01 ± 0.19 mm and 0.21 ± 0.04 mm^2^) than in the post-phaco group (0.75 ± 0.21 mm, and 0.15 ± 0.04 mm^2^) (*P* < 0.01, *P* < 0.05, respectively). Although the difference was not significant, CCW in the normal eye group (0.57 ± 0.18 mm) was larger than that in the post-phaco group (0.52 ± 0.15 mm) (*P* = 0.42) (Figures 4A–C). These results indicate that the CC is smaller in post-phaco group compared with that in the normal eye.

No significant differences were observed in AOD and ICA when comparing all groups (Figures 4D, E). These results suggest that cataract progression and phacoemulsification did not have significant effects on these factors in dogs.

## 4 Discussion

In this clinical study, we employed UBM to examine the differences in the CC among normal eyes, cataract eyes, and eyes after phacoemulsification. The CC is known to play a critical role in the outflow of AH and serves as a shared pathway for both conventional and unconventional routes (5). Despite the significance of the CC in AH dynamics, there is a paucity of research investigating alterations in this anatomical region in dogs.

Previous studies have attempted to identify POH in dogs by observing the ICA using UBM. Rose et al. (11) found that an ICA >13°, measured before surgery, could be a predisposing factor for POH. Crumley et al. (23) reported a weak correlation between the AOD measured before phacoemulsification and POH. However, these studies did not focus on ICA changes but rather on predisposing factors before surgery. Furthermore, they did not analyse the causes of glaucoma, one of the most serious complications after phacoemulsification, but only POH, which typically occurs within 2 h after surgery and resolves within 24 h (15, 16, 24). In contrast to previous studies, the main focus of our study was to investigate the differences in the CC between eyes with cataracts and eyes that underwent phacoemulsification. In addition, unlike previous studies that examined acute changes after phacoemulsification, we investigated the CC changes by targeting canine patients which underwent surgery at least 2 months prior (662 ± 465 days, range: 61–1,365 days). This is a significant point of difference.

To further investigate the differences in AH outflow in dogs after phacoemulsification, we employed specific CC parameters, namely CCW, CCL and CCA. To ensure the validity of these parameters, we excluded cases with closed ICA because of the difficulty in observing these areas (17). Patients with post-operative glaucoma occurring after surgery were excluded from the study. This is because previous studies have shown that the collapse of the CC occurs in cases of glaucoma, which was unsuitable for this study (18).

In this study, various measures were undertaken to guarantee the consistent state of each subject. First, tropicamide was uniformly administered across all groups, ensuring equal iris dilation at the time of the UBM measurement. Although pupil diameter was not specifically measured in this research, the application of tropicamide guaranteed consistent and full dilation prior to the measurement, thereby eliminating intergroup dilation differences. This uniform application was intended to reduce variability in the measured parameters, attributed to differences in light-induced miosis. This methodology is supported by the research of Thomas Dulaurent et al., which highlights the alteration in CC parameters due to mydriasis induced by tropicamide (19). Secondly, the measurement location for UBM was consistently maintained in the dorsal quadrant. This decision was informed by existing practices in human medicine, where dividing the eye into temporal, nasal, superior, and nasal sections showed no significant difference in AOD, trabecular-ciliary process distance and trabecular-iris angle between sections (20). Additionally, the dorsal position was the most visible when dogs were in a sitting position, leading to consistent measurements at this location. Lastly, diabetic dogs were excluded from this study. According to research by Wilkie et al., out of 40 eyes affected with cataracts, 30 had a spontaneous rupture of the lens capsule prior to surgery, which could affect the CC by inducing LIU. Moreover, the study excluded cases with intumescent cataracts prevalent in diabetic conditions, which could exert a more considerable force on the lens zonule, potentially impacting the CC (25).

In this study, the CCW, CCL, and CCA in the candidate phacoemulsification group were significantly larger than those in the non-candidate group. The difference of the CC can be explained by a study on presbyopia in humans (26). A previous study reported that the hardening of the crystalline lens caused by cataracts could influence the contractility of the ciliary muscle in humans, which can be related to the CC in dogs (26). Moreover, the presence of cataracts induces pathological changes in the lens, resulting in increased lens thickness. This anatomical alteration subsequently leads to heightened zonular tension, which in turn keeps the ciliary muscle in a relaxed state (26–28). During relaxation, the ciliary muscles moved in the posterior and outward direction (29). Consequently, due to these pathological changes, the candidate group exhibited relatively larger values of CCW, CCL, and CCA compared to the non-candidate group. This phenomenon is consistent with our finding that cataract maturation extends into the CC.

In addition, as the cataracts matured, CCW, CCL, and CCA tended to increase. Based on these results, we confirmed that as the cataract stage increased, the ciliary muscle relaxed and the CC expanded. The effect on the zonules and ciliary muscles increased as the cataracts progressed. This can be explained by an increase in lenticular sclerotic components with increasing cataract severity.

In this study, we found that the CCW, CCL, and CCA levels became smaller after phacoemulsification. In addition, CCA and CCL were significantly larger in the normal eye group than in the post-phaco group. These results are consistent with those of a previous study showing CC narrowing after experimental phacoemulsification in non-cataractous, normal experimental dog eyes. However, in a previous study, as the difference in our study, non-cataractous normal eyes were used, and the CC were measured using histological examination at 3 and 24 h after phacoemulsification (30). The previous studies had some limitations. First, during histological tissue processing and block sectioning, there is a possibility of anatomical alterations, including anterior chamber narrowing (31). Second, uveitis occurs in 90% of non-diabetic dogs immediately after phacoemulsification (1, 32). Prostaglandin mediators produced in uveitis can affect CC parameters (33, 34). One study reported that the incidence of CCA increased when latanoprost, a prostaglandin mediator, was used alone (33). Therefore, in our study, to overcome these limitations, UBM was used to non-invasively visualize living tissues without mechanical or chemical deformation of the anterior chamber (7, 35–37). Moreover, to eliminate the possible effects of uveitis soon after phacoemulsification, only eyes without inflammation for more than 2 months after phacoemulsification were selected.

Regarding the short-term results of phacoemulsification, a previous report hypothesized that released tension in the lens zonule and the use of atropine for cycloplegia after phacoemulsification may result in peripheral movement of the ciliary body and compression of the TM (30). However, in our study, cases at least 2 months after phacoemulsification were selected (i.e., not immediately after surgery), and atropine was not used for at least 6 weeks before the UBM test; therefore, the effect of atropine on CC could be excluded.

According to previous studies on the contractility of the ciliary muscle in humans, when 2% pilocarpine was instilled to contract the ciliary muscle in eyes with cataracts before phacoemulsification, the movement of the ciliary body was rigid (26). However, after phacoemulsification, the tension in the zonule was reduced and ciliary body movement improved. This indicated that the ciliary muscles remained in a relatively contracted state. During contraction, the ciliary muscle moves in the anterior and inward directions (29). This movement of the ciliary muscle causes the CC to narrow. In human studies, an increase in the contractility of the ciliary body after phacoemulsification can lead to narrow of the CC. In addition, surgical procedures such as I/A, IOL implantation, and flattening of the lens after phacoemulsification may have affected CC collapse.

The use of ICA and AOD seems inappropriate in veterinary medicine because of differences in measurement methods and anatomy. First, the measurement method lacks consistency and there are no clear anatomical landmarks, which results in a wide range of ICA and AOD values in dogs. This variability makes it difficult to establish reliable benchmarks, as previous research has shown no significant differences in the ICA and AOD before and after surgery in dogs (11). In addition, consistent landmarks such as the scleral spur in humans do not exist in dogs because of anatomical differences, leading to several inconsistent methods for measuring AOD and ICA in veterinary medicine (38–41). This variability in measurement methods can induce further inconsistencies in ICA and AOD values in dogs. Second, the anatomical differences between humans and dogs limit the use of the AOD and ICA in the same way in veterinary medicine. For example, the TM is situated within the recess of the CC in dogs (5, 12, 42), whereas in humans, it is located anteriorly relative to the root of the iris and sits within the scleral sulcus (43–45). Consequently, iris dilation in humans can obstruct access to the TM by causing external and anterior displacement of the iris root (46, 47). However, the location of the TM in dogs makes it unlikely that movement of the iris root would affect the outflow of the AH. Therefore, AOD and ICA may not be as significant in veterinary medicine as they are in human medicine because they reflect iris movement, which is most affected by these anatomical differences. For these reasons, geometric factors such as AOD and ICA may not be appropriate in veterinary medicine, unlike human medicine, owing to differences in measurement methods and anatomy. Instead, CC parameters such as CCW, CCL, and CCA, which reflect anatomical factors in dogs, may be more appropriate.

This study had some limitations. First, it was a comparison between groups and not an assessment before and after phacoemulsification in the same dogs. Although the sample size was small (*n* = 4), the same results as those in this study were obtained in the comparison before and after phacoemulsification in the same dogs (data not shown). In addition, there was no significant difference in body weight between the groups. Second, narrowing of the CC did not directly cause glaucoma in any of the dogs in our study. Because of the compensatory pathway of IOP, which adjusts the production and outflow of AH according to the change in IOP, IOP tends to return to near baseline (48). However, the narrowing of the CC may contribute to discordance in the compensatory mechanism (19). Therefore, the narrowing of the CC may be an important anatomical factor in canine glaucoma after phacoemulsification.

Furthermore, in human medicine, it is recognized that the use of steroid medication can potentially precipitate glaucoma. This phenomenon, termed steroid-induced glaucoma, is categorized as a form of secondary open-angle glaucoma initiated by augmented resistance to aqueous outflow at the trabecular meshwork level. This condition is characterized by the increased production and diminished breakdown of the extracellular matrix of the trabecular meshwork (49). Previous studies have identified the immediate postoperative application of topical flurbiprofen plus corticosteroids as a potential predisposing factor for glaucoma development following cataract surgery in dogs. However, the solo use of steroids is not reputed to elevate glaucoma risk significantly (50). In this study, only steroids were used, and all medication effects were eradicated by measuring UBM 2 months post-phacoemulsification, mitigating the influence of this factor on the results.

Future studies are warranted based on these findings. The influence on CC narrowing can be studied according to the duration of I/A, volume of BSS, phacoemulsification power, and time. In addition, studies on prophylactic medications that reduce AH production, such as beta-blockers and carbonic anhydrase inhibitors, may be helpful.

In summary, as cataracts occur, the CC expands compared to normal eyes. After phacoemulsification, the CC narrowed more than in normal eyes (Graphical Abstract). The CC loses its normal flexibility after cataract formation and phacoemulsification. A narrowed CC can increase AH resistance. In conclusion, anatomical narrowing of the CC after phacoemulsification of the cataractous eye is a possible cause of glaucoma in dogs.

## Data availability statement

The original contributions presented in the study are included in the article/supplementary material, further inquiries can be directed to the corresponding author.

## Ethics statement

The animal studies were approved by Chungbuk National University Institutional Animal Care and Use Committee (CBNUA-1700-22-02). The studies were conducted in accordance with the local legislation and institutional requirements. Written informed consent was obtained from the owners for the participation of their animals in this study.

## Author contributions

DK made substantial contributions to the conception or design of the work, analysis, and interpretation of data and also played a significant role in drafting the work and revising it critically for important intellectual content. Y-SG and HK were responsible for examining the study participants and assessing their suitability for the research. S-EP, JH, NK, and JJ were involved in collecting data from the study participants. All authors contributed to the article and approved the submitted version.
